# Prevalence and Correlates of Problematic Alcohol Use Among Older Chinese Adults: A Study Combining Logistic Regression and Psychological Network Analysis

**DOI:** 10.31083/AP45934

**Published:** 2026-04-21

**Authors:** Hang Qian, She-Hui Chang, Bao-Liang Zhong

**Affiliations:** ^1^Department of Psychiatry, Affiliated Wuhan Mental Health Center, Tongji Medical College of Huazhong University of Science and Technology, 430030 Wuhan, Hubei, China; ^2^Research Center for Psychological and Health Sciences, China University of Geosciences (Wuhan), 430074 Wuhan, Hubei, China

**Keywords:** Chinese population, older adults, problematic alcohol use, depression, network analysis

## Abstract

**Background:**

Alcohol use inflicts substantial harm on older adults; however, research in China remains limited. The association between alcohol use and depression at the symptom level is particularly unclear. This study examined the prevalence and correlates of problematic alcohol use (PAU) and the network structure of comorbid PAU and depressive symptoms among older Chinese adults.

**Methods:**

A cross-sectional survey was conducted with 2643 adults aged ≥60 years in Wuhan, China. PAU was assessed using the Alcohol Use Disorders Identification Test (AUDIT), and depressive symptoms were measured with the 9-item Patient Health Questionnaire (PHQ-9). Multivariate logistic regression identified correlates of PAU. A regularized partial correlation network was constructed to examine the comorbid structure of AUDIT and PHQ-9 symptoms.

**Results:**

The prevalence of any alcohol use was 21.0%, and that of PAU was 8.4%. Significant correlates of PAU included depressive symptoms (OR = 1.68, *p* = 0.004), male sex (OR = 2.87, *p* = 0.001), employment (OR = 1.51, *p* = 0.034), rural residence (OR = 1.51, *p* = 0.034), and lower monthly household income (0–56 USD vs. ≥490 USD: OR = 4.02, *p* = 0.020). In the network, the strongest edges were AUDIT–PHQ-1 “anhedonia” (edge weight = 0.272) and AUDIT–PHQ-3 “sleep disturbance” (edge weight = 0.183). Centrality indices revealed that PHQ-3 “sleep disturbance” and PHQ-1 “anhedonia” were the most central symptoms.

**Conclusions:**

Depressive symptoms were strongly associated with PAU among older Chinese adults. Male sex, employment, rural residence, and low income increased the risk. Anhedonia and sleep disturbance emerged as pivotal depressive symptoms bridging PAU. Interventions targeting reward processing and sleep regulation may mitigate this comorbidity.

## Main Points

1. The prevalence of alcohol use and problematic alcohol use (PAU) among older adults was 21.0% and 8.4%, respectively.

2. The factors significantly associated with PAU were male sex, low income, employment status, and depressive symptoms.

3. This study is the first to adopt psychological network analysis to map the symptom-level comorbidity structure between PAU and depression in older adults.

4. Network analysis indicated that multiple depressive symptoms are directly associated with PAU. Among these, anhedonia and sleep disturbance are most closely linked to PAU, serving as the core symptoms of the network.

5. Relevant stakeholders should enhance monitoring and interventions for elderly people who are experiencing specific social risks. Interventions targeting reward activation and circadian rhythm regulation hold promise as effective pathways to break the reinforcing cycle between depression and PAU.

## 1. Introduction

Alcohol use ranks among the leading risk factors contributing to the global burden of disease [1, 2, 3, 4]. The current evidence demonstrates that no level of alcohol consumption confers clear health benefits, whereas excessive intake substantially elevates risks for numerous adverse health outcomes, particularly in older adults [4, 5]. As China undergoes profound population aging [6], alcohol use in this demographic and its associated health consequences represent a growing public health challenge [7]. Epidemiological data indicated that, over the past decade, the drinking rate among older Chinese adults has ranged from 27.3% to 52.5% [8, 9, 10, 11, 12], with a current average drinking rate of 28.2% [13].

Previous studies have considered problematic alcohol use (PAU), including hazardous, potentially hazardous, risky, at-risk, harmful, problematic, or heavy drinking or alcohol use, as well as alcohol misuse, alcohol dependence, alcohol use exceeding the guidelines and alcohol use disorder [14, 15, 16]. The World Health Organization (WHO) estimates that the prevalence of harmful alcohol use among Chinese adults aged ≥60 years is 11.4% [17]. A recent nationwide survey reported a hazardous drinking rate of 9.1% in this population [18]. Collectively, these findings underscore that alcohol consumption and associated problems are prevalent among older adults in China and warrant further investigation.

The adverse effects of alcohol use are particularly pronounced in older adults and have substantial clinical and public health implications [19]. Physiologically, age-related declines in metabolic capacity, reduced lean body mass, and prevalent polypharmacy diminish alcohol tolerance and heighten sensitivity. Consequently, equivalent alcohol intake yields higher blood alcohol concentrations in older adults, markedly increasing the risks of falls, accidental injuries, liver disease, cardiovascular disorders, and neurological impairment (such as Wernicke-Korsakoff syndrome and alcoholic dementia) [20, 21]. Psychologically, alcohol use can exacerbate preexisting mental health conditions such as depression and anxiety, while also precipitating emotional dysregulation and cognitive decline, thereby profoundly impairing the quality of life of older adults [12, 22, 23].

Prior research has examined correlates of PAU in older populations across demographic, sociological, and psychological domains. Demographic and sociological factors associated with elevated PAU risk include male sex, rural residence, lower socioeconomic status, and unemployment [24, 25]. Retirement or job loss, in particular, may precipitate stress, loneliness, and diminished self-worth, prompting older adults to use alcohol as a maladaptive coping strategy [26, 27, 28]. Substantial evidence indicates that psychosocial stress heightens alcohol cravings and intake, playing a pivotal role in the etiology of alcohol dependence [29, 30, 31].

Among the psychological factors associated with PAU, depression plays a particularly central role [32, 33, 34, 35]. Late life represents a high-risk period for depressive disorders [36, 37, 38]. Owing to its sedative and euphoric properties, alcohol is frequently employed as a maladaptive strategy to mitigate negative affect, including depression and anxiety [39, 40]. This pattern comports with the “self-medication” hypothesis, whereby individuals consume alcohol to palliate unmet psychological needs or untreated emotional distress. This hypothesis, first proposed by Khantzian EJ [41, 42], posits that individuals, when facing emotional pain, psychological distress, or loss of life roles (such as retirement, unemployment, or a reduction in social roles), may choose alcohol or other substances to “self-medicate” or alleviate negative emotions [26]. However, such coping is ultimately counterproductive: chronic alcohol neurotoxicity impairs cerebral function and intensifies depressive symptomatology, engendering a pernicious, self-perpetuating cycle [43, 44].

Although the extant research provides a foundation for understanding PAU among older adults [45, 46], notable limitations persist. First, systematic investigations focused on older Chinese adults remain scarce, with a particular dearth of contemporary epidemiological data on PAU patterns amid evolving socioeconomic conditions. Second, methodologically, prior studies have predominantly employed conventional regression models (e.g., logistic regression) to delineate associated factors. Although these approaches quantify overall associations, they inherently treat depression and PAU as unitary, homogeneous constructs—typically operationalized via total scores or dichotomous indicators—thereby obscuring the nuanced interplay among constituent symptoms [47]. This “black-box” paradigm precludes answers to questions critical for precision intervention, such as which specific depressive symptoms (e.g., anhedonia) most strongly drive PAU.

To address these gaps, this study pursues two primary objectives while emphasizing its methodological innovation. First, we conducted a large-scale cross-sectional survey in a major Chinese city to provide contemporary data on the prevalence of PAU and its correlates among older adults. Second, and most notably, we applied psychological network analysis to elucidate symptom-level interconnections between depressive symptoms and PAU. Network theory posits that psychopathology emerges from dynamic interactions among symptoms rather than a singular latent cause [48]. This approach has gained prominence for mapping psychopathological symptom interplay, particularly in depression research. Applications include examining symptom-level links between suicidal ideation and comorbid depression-anxiety in Chinese adolescents [49], and associations between severe depressive symptoms and low-grade inflammation [50]. Network analysis can be integrated with traditional association methods, such as logistic regression and least absolute shrinkage and selection operator (LASSO) regression, to identify and explore key risk factors for diseases as well as the complex interrelationships among them [51, 52, 53, 54]. These studies have yielded critical insights. By constructing a symptom network, we visually identified central nodes within the comorbid depression-PAU structure [55]. This paradigm shift—from mere correlation to mechanistic connectivity—illuminates pathways sustaining comorbidity, thereby informing targeted prevention and intervention strategies [56, 57].

## 2. Materials and Methods

### 2.1 Participants 

This study was conducted between March and September 2024 in Wuhan, a megacity in central China. A multistage stratified sampling method was employed to ensure representativeness across urban, suburban, and rural–urban fringe areas. Stratification was based on administrative districts and socioeconomic levels. Eight communities were randomly selected as survey sites. Within each community, older adults undergoing routine annual health examinations at community health centers were consecutively invited to participate until the target quota was reached.

The inclusion criteria were: age ≥60 years; continuous residence in the selected community for ≥6 months prior to the survey; and voluntary participation with written informed consent. The exclusion criteria were: severe physical illness (e.g., end-stage cancer, cardiovascular and cerebrovascular diseases, and other serious diseases that result in an inability to respond); cognitive impairment precluding questionnaire comprehension or response (based on previous medical records and family-reported information), and an inability to provide informed consent. All the participants completed the questionnaire under the guidance of trained investigators. For participants who could not understand written Chinese, their family members used local languages to assist our researchers in completing the survey. All participants were provided with detailed information about the specific procedures of this study prior to providing informed consent. They completed the questionnaire anonymously and received a gift prepared by the community upon completion of the study.

In our pilot study, the prevalence of suspected PAU was 6.9% in a small sample of 50 older adults in Wuhan. Accordingly, the parameters used for the sample size estimation were set as follows: a 6.9% prevalence, a 0.025 confidence interval (CI) width, a two-sided 0.05 type I error rate, and an 80% response rate. By using the formula for sample size estimation for cross-sectional studies, the minimum sample size needed was 1984 [58]. According to a widely accepted principle in network analysis, the minimum sample size should be at least 10 times the number of nodes (i.e., N ≥10p). The minimum sample size for the present study was 120. A total of 2661 questionnaires were collected. The proportion of missing values across all variables included in the analysis was less than 5%. First, 18 cases with more than four missing values for key variables were removed. Given the very low overall missingness rate, the remaining missing values were imputed using mode imputation. This approach preserves the categorical nature of the variables and introduces minimal distortion to the data distribution. After data cleaning and imputation, the final analytic sample consisted of 2643 valid cases.

### 2.2 Variables

#### 2.2.1 Problematic Alcohol Use

Alcohol use was assessed using the Chinese version of the Alcohol Use Disorders Identification Test (AUDIT), with total scores ranging from 0 to 40. A score greater than 7 was considered indicative of PAU [59]. The Chinese version of the AUDIT questionnaire has demonstrated high validity in local Chinese studies [60]. In this study, the Cronbach’s α coefficient of the Chinese AUDIT was 0.867, reflecting good internal consistency.

#### 2.2.2 Depressive Symptoms

Depressive symptoms were measured using the Chinese version of the Patient Health Questionnaire-9 (PHQ-9), a widely used screening tool for clinically significant depressive symptoms with good reliability and validity among older adults in China [61]. Each item is rated on a 4-point scale ranging from 0 (“not at all”) to 3 (“nearly every day”). The Cronbach’s α coefficient for the PHQ-9 in this study was 0.884, indicating high internal consistency. A total score greater than 4 was considered indicative of depressive symptoms.

#### 2.2.3 Perceived Stress

Perceived stress was evaluated using the Perceived Stress Scale (PSS), which assesses the degree to which life situations are appraised as unpredictable, uncontrollable, or overwhelming [62]. Each item is rated on a 5-point Likert scale from 0 (never) to 4 (very often), with total scores ranging from 0 to 40, with higher scores reflecting greater perceived stress. The PSS has demonstrated good reliability and validity in Chinese populations [63]. In this study, the Cronbach’s α coefficient was 0.762. Perceived stress was defined as a score above the median PSS score of 27 in this sample.

#### 2.2.4 Other Variables

Sociodemographic variables were collected via structured interviews and included: sex, age, marital status (married vs. unmarried), educational attainment (illiterate, below college, college degree or higher), average monthly household income (0–56 USD, 56–140 USD, 140–280 USD, 280–490 USD, ≥490 USD), employment status (employed vs. unemployed), and residence (urban vs. rural).

### 2.3 Statistical Analysis

All the statistical analyses were performed using R version 4.4.3 (https://cran.r-project.org/bin/windows/base/old/4.4.3/). Statistical significance was defined as a two-sided *p* < 0.05. Correlates of PAU were examined in two stages, univariate analyses followed by multivariate logistic regression. Sociodemographic characteristics, perceived stress, and depressive symptoms were compared between participants with and without PAU using chi-square or rank-sum tests, as appropriate. Variables significant in the univariate analyses were entered into a multivariate logistic regression model to identify independent correlates of PAU. Backward elimination based on the Wald χ^2^ statistic was used for model selection. Odds ratios with 95% confidence intervals (CIs) were computed to estimate the strength of the associations.

To examine symptom-level associations between depressive symptoms and PAU, we estimated a psychological network comprising individual PHQ-9 items and the AUDIT total score. Key covariates identified in the multivariate logistic regression were included as nodes to adjust for confounding and enhance the precision of edge estimates. Given the inclusion of both continuous (PHQ-9 items, AUDIT total) and categorical variables (covariates), a mixed graphical model (MGM) was estimated using the estimateNetwork function from the bootnet package (version 1.6) in R. Regularization was applied via LASSO, which sets trivial edge weights to zero, yielding a sparse and interpretable network, and has been widely applied to psychological network estimation. The tuning hyperparameter was fixed at γ = 0.25 to balance sensitivity and specificity. The network graph was constructed using the qgraph package (version 1.9.8). The node layout adopted a “spring” pattern, retaining all nonzero edges to display all associations.

Node centrality was quantified using three indices: strength, betweenness, and closeness. Strength represents the sum of absolute edge weights directly connecting a node to others, reflecting direct influence. Betweenness measures the frequency with which a node lies on the shortest path between pairs of other nodes, indicating bridging importance. Closeness quantifies the inverse of the average shortest path length to all other nodes, capturing the indirect influence and efficiency of propagation [64]. These metrics collectively identify the most central, interconnected, and influential symptoms in the network.

Network accuracy and stability were evaluated using the bootnet package. Edge-weight accuracy was assessed via nonparametric bootstrapping (1500 iterations) to derive 95% confidence intervals. Stability was examined using case-dropping bootstrapping with the correlation stability (CS) coefficient, for which values ≥0.25 were deemed acceptable, while values >0.5 indicated excellent stability. Additionally, bootstrapped difference tests were conducted for edge weights and centrality indices to evaluate structural robustness.

## 3. Results

### 3.1 Demographic Characteristics

A total of 2643 participants (men/women: 1296/1347, age: 69.9 ± 7.0 years, range: 60–97 years) completed the survey questionnaire. Among the total sample surveyed, 555 (21.0%) reported the habit of consuming alcoholic beverages. A total of 222 participants had an AUDIT score ≥7, yielding a PAU prevalence of 8.4%. Sample characteristics of participants are presented in Table 1.

**Table 1.** **Sociodemographic characteristics of the participants**.

Variable		N	Problematic alcohol use	χ^2^/H	*p*
Sex					
	Male	1296	161 (12.4%)		
	Female	1347	61 (4.5%)	53.499	<0.001
Age (years)					
	≥75	364	20 (5.5%)		
	60–74	2279	202 (8.9%)	4.630	0.040
Marital status					
	Married	2311	200 (8.7%)		
	Unmarried	332	22 (6.6%)	1.551	0.213
Education level					
	Illiterate	330	29 (8.7%)		
	Below college	2038	169 (8.7%)		
	College degree and above	275	24 (8.7%)	0.133	0.936
Employment status					
	Employed	282	41 (14.5%)		
	Unemployed	2361	181 (7.7%)	15.465	<0.001
Average monthly income (USD)					
	0–56	501	80 (16.0%)		
	56–140	466	38 (8.2%)		
	140–280	916	66 (7.2%)		
	280–490	618	32 (5.2%)		
	>490	142	6 (4.2%)	50.567	<0.001
Place of residence					
	Urban	1437	81 (5.6%)		
	Rural	1206	141 (11.7%)	31.243	<0.001
Depressive symptoms					
	No	1822	101 (5.5%)		
	Yes	821	121 (14.7%)	13.184	<0.001
Perceived stress					
	No	1188	128 (10.8%)		
	Yes	1455	94 (6.5%)	0.665	0.415

### 3.2 Potential Covariates of PAU

The results of univariate analysis (Table 1) indicate that men, older adults aged 60–74 years, those who were employed, those with a monthly income of 0–56 USD, rural older adults, and depressed older adults had a statistically higher prevalence of PAU than their corresponding counterparts did (*p* ≤ 0.040).

### 3.3 Significant Correlates of PAU

As displayed in Table 2, the factors significantly associated with PAU among Chinese older adults were depressive symptoms (OR = 1.68, *p* = 0.004), male sex (vs. female, OR = 2.87, *p* = 0.001), employment (vs. unemployment, OR = 1.51, *p* = 0.034), and a lower monthly household income (for the 0–56 USD group, OR = 4.02, *p* = 0.020, compared to the ≥490 USD reference group).

**Table 2.** **Significant correlates of problematic alcohol use among older adults: findings from multiple logistic regression analysis**.

Variables		OR (95% CI)	*p*
Depressive symptoms			
	Yes (vs. no)	1.68 (1.19, 2.38)	0.004
Sex			
	Male (vs. female)	2.87 (2.10, 3.92)	0.001
Employment			
	Employed (vs. unemployed)	1.51 (1.03, 2.21)	0.034
Average monthly income (USD)			
	≥490 (reference group)	1	
	0–56	4.02 (1.70, 9.51)	0.020
	56–140	2.11 (0.87, 5.14)	0.100
	140–280	1.85 (0.78, 4.37)	0.164
	280–490	1.25 (0.51, 3.08)	0.623

CI, confidence interval.

### 3.4 Network Structure of PHQ-9 and PAU

**Fig. 1.**
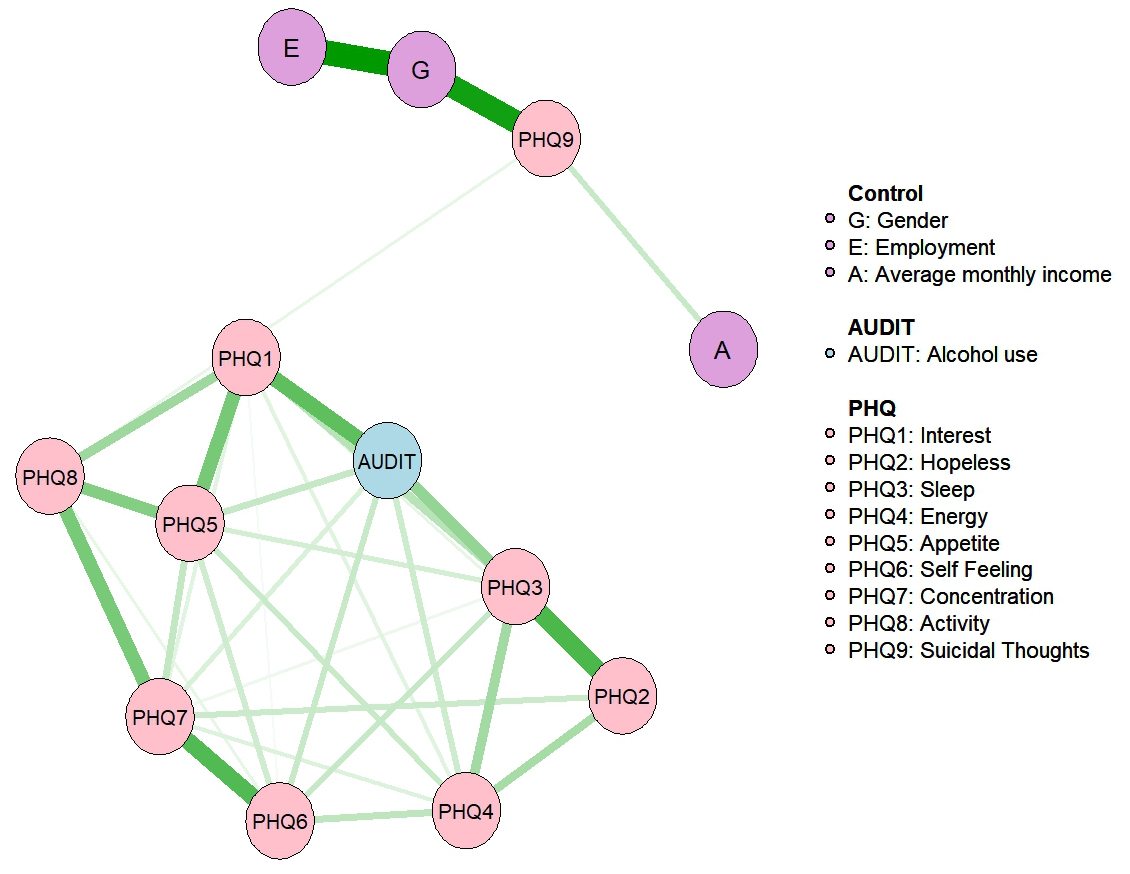
**Network of PHQ-9 and PAU**. PHQ-9, 9-item Patient Health Questionnaire; PAU, problematic alcohol use.

Fig. 1 presents the estimated network comprising PHQ-9 items, the AUDIT total score, and selected demographic covariates (sex, educational attainment, and per capita income). It shows 34 nonzero edges out of 78 possible edges. The AUDIT node exhibited robust connections with multiple depressive symptoms, with the strongest edges linking AUDIT to the PHQ-1 “anhedonia” (edge weight = 0.272) and the PHQ-3 “sleep disturbance” (edge weight = 0.183). No direct edges connected demographic covariates to AUDIT scores. Within the depressive symptom cluster, the edge between PHQ-2–PHQ-3 (depressed mood–sleep disturbance; edge weight = 0.305) and that between PHQ-6–PHQ-7 (guilt–concentration difficulties; edge weight = 0.295) were the strongest associations.

### 3.5 Network Centrality 

**Fig. 2.**
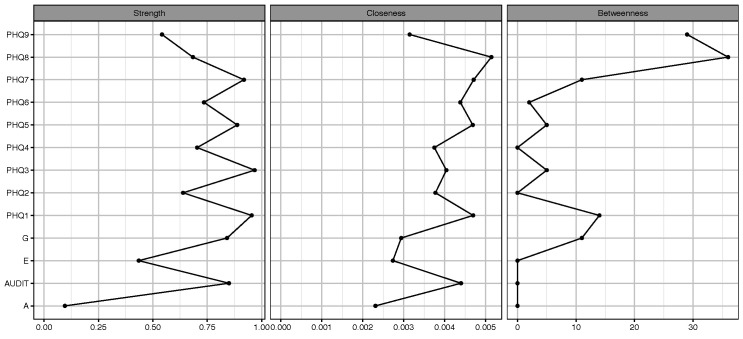
**Centrality of network nodes**.

Centrality analysis (Fig. 2) identified that PHQ-3 “sleep disturbance” (strength = 0.968, betweenness = 5, closeness = 0.004) and PHQ-1 “anhedonia” (strength = 0.954, betweenness = 14, closeness = 0.005) were the most core symptoms.

### 3.6 Network Predictability and Stability 

**Fig. 3.**
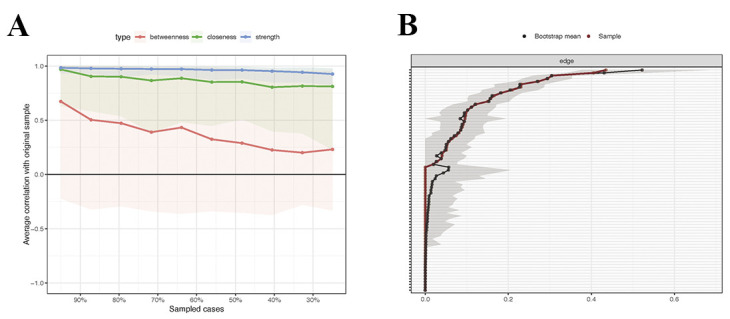
**Stability and Accuracy of the network**. Notes: (A) Stability of centrality using case-drop bootstrapping. (B) Accuracy of edge weights using case-drop bootstrapping.

Network stability was evaluated using the correlation stability coefficient (CS-coefficient). Case-dropping bootstrapping revealed high stability for node strength (CS-coefficient = 0.75; Fig. 3A). Non-parametric bootstrapping (1500 iterations) yielded narrow 95% confidence intervals around most edge weights, indicating high estimation precision (Fig. 3B).

## 4. Discussion

This study revealed a prevalence of PAU of 8.4%, with male sex, lower household income, employment, and depressive symptoms emerging as significant correlates. Network analysis demonstrated direct associations between multiple depressive symptoms and PAU, with anhedonia and sleep disturbance exhibiting the strongest connections and serving as the most central nodes in the comorbid network.

### 4.1 Interpretation of Sociodemographic and Psychosocial Correlates

The observed PAU prevalence of 8.4% in this study is a significant public health concern. Although estimates vary because of differences in assessment methods and diagnostic thresholds, this figure aligns with accumulating evidence indicating that alcohol use disorders constitute a significant and escalating issue in China [65, 66]. Notably, older adults are not exempt from this trend. Moreover, the true burden may be underestimated, as substance use disorders in geriatric populations are frequently underrecognized and misattributed to normative aging processes, polypharmacy effects, or comorbid medical conditions—particularly when symptoms such as cognitive decline, sleep disturbances, or functional impairment overlap with age-related changes [67, 68, 69]. In light of global evidence that the “Baby Boomer” cohort exhibits higher lifetime substance use than prior generations do [70], this 8.4% prevalence should be regarded as a sentinel indicator of a broader, often concealed public health challenge—one likely to intensify with ongoing population aging. A brief intervention approach targeting alcohol use may play a role in preventing the development of alcohol use disorders among elderly people [71].

Multivariate logistic regression confirmed several established risk factors for PAU. Male sex was associated with nearly threefold greater odds of PAU (OR = 2.87), which is consistent with extensive evidence from China and internationally identifying male sex as the strongest demographic predictor of alcohol-related problems [72, 73]. This disparity likely reflects entrenched sociocultural norms governing gender-specific drinking behaviors. Similarly, lower household income—particularly the lowest bracket (OR = 4.02)—emerged as a significant risk factor, corroborating prior research [74, 75]. Individuals in lower socioeconomic strata typically experience heightened psychosocial stress, adhere to distinct cultural drinking practices, and face limited access to health care and adaptive coping alternatives.

Notably, being employed emerged as a significant correlate for PAU (OR = 1.51), which contrasts with much of the Western literature that links unemployment or retirement stress to increased alcohol use [25]. This discrepancy is due to the explicable within the distinctive sociocultural role of alcohol in Chinese professional settings. In China, heavy drinking is rarely solitary or pub-centered; rather, it is deeply embedded in relational and occupational rituals [76]. Alcohol serves as a key medium for cultivating *guanxi*, demonstrating respect, and sealing business transactions. For employed older adults—particularly those in managerial, advisory, or client-facing positions—this entails recurrent, often obligatory exposure to high-volume drinking. Thus, risk arises not from unemployment-related distress but from culturally sanctioned drinking norms tied to occupational roles. This observation underscores the imperative role of cultural context in risk factor interpretation and illustrates how a variable protective in one sociocultural milieu may confer vulnerability in another.

In contrast to prior evidence linking stress to alcohol consumption, perceived stress was not significantly associated with PAU in the final multivariate model. This null finding does not refute the role of stress but warrants nuanced interpretation. One explanation is enhanced resilience in older adults, who may have cultivated adaptive, nonsubstance coping strategies over the life course—such as leveraging social support or engaging in communal activities—to manage stressors [77]. Alternatively, measurement limitations may account for the absence of an association. The Perceived Stress Scale (PSS) provides a global assessment of stress and may insufficiently capture chronic, age-specific stressors that are salient to older adults, including bereavement, chronic illness management, or functional decline. The instrument may be more attuned to acute, work-related, or psychosocial stressors prevalent in younger cohorts. Thus, the null result could reflect a mismatch between the tool and the phenomenological landscape of late-life stress. Future studies should incorporate domain-specific stress measures to more accurately delineate stress–alcohol dynamics in older populations.

### 4.2 Deconstructing the Depression-PAU Comorbidity: A Network Perspective

Network analysis revealed that, at the micro level, sociodemographic factors (sex, employment, income) indirectly influence PAU via depressive symptoms. Depressive symptoms were positively associated with PAU. Anhedonia (PHQ-1) and sleep disturbance (PHQ-3) displayed not only the strongest direct edges to PAU but also the highest centrality indices within the network. These results indicate that anhedonia and sleep disturbance function as core pathological nodes bridging depression and PAU in older adults.

Anhedonia, a cardinal feature of depression, reflects dysfunction in the reward circuitry of the brain [78, 79]. It is closely linked to reduced dopaminergic activity in the nucleus accumbens and ventral tegmental area (VTA) [80]. Age-related atrophy and diminished reward responsivity in these regions contribute critically to late-life depression [81, 82, 83]. According to the self-medication hypothesis, persistent anhedonia and loss of interest may drive individuals to seek transient reward restoration through exogenous substances, such as alcohol [41]. Alcohol acutely enhances dopamine release—particularly in the ventral striatum—producing temporary mood elevation and pleasure reinstatement, thereby reinforcing drinking behavior [84, 85]. Chronic exposure, however, induces dopamine receptor desensitization, escalating cravings and fostering dependence. Thus, anhedonia likely serves as a pivotal pathological relay in the depression–PAU network. Interventions targeting the reward system—such as behavioral activation [86], therapies targeting the positive system (such as increasing valuable activities, rebuilding reward expectations, and monitoring positive reinforcement pathways), and reward-oriented cognitive therapy—are effective measures for improving anhedonia [87] and have demonstrated efficacy in alleviating anhedonia. These approaches may represent critical therapeutic strategies for restoring reward function in older adults and interrupting the bidirectional reinforcement between depression and alcohol dependence.

Sleep disturbance (PHQ-3) also emerged as a prominent node strongly associated with PAU in the network. Compared to younger adults, older adults are more vulnerable to insomnia and exhibit prolonged sleep latency, reduced REM sleep duration, and markedly lower sleep efficiency [88, 89]. In depression, circadian dysregulation—manifesting as delayed melatonin secretion and HPA axis hyperactivity—further exacerbates sleep disruption [90, 91]. Thus, in late life, sleep disturbance may represent a core phenotypic expression of depression with significant physiological underpinnings. Previous experimental studies have indicated that alcohol has complex, multifaceted effects on sleep architecture: acutely, it promotes sleep initiation via sedation but suppresses REM and deep sleep stages [92], and chronically, it induces pervasive sleep fragmentation, prolonged latency, diminished efficiency, and reduced REM duration—patterns that mirror and amplify depressive sleep pathology [93]. This bidirectional interplay positions sleep disturbance as a key physiological conduit in depression–PAU comorbidity among older adults. Early intervention targeting sleep represent a crucial entry point for disrupting this cycle. Foundational approaches include sleep hygiene education and increased physical activity. Cognitive behavioral therapy for insomnia (CBT-I) and geriatric-appropriate pharmacotherapy have demonstrated efficacy in ameliorating insomnia [94, 95, 96]. Such interventions not only alleviate depressive symptoms but also attenuate alcohol cravings and reduce relapse risk, offering a dual-benefit pathway for managing comorbidities in this population.

### 4.3 Limitations

Several limitations of this study merit consideration. First, the cross-sectional design precludes causal inference; longitudinal studies are needed to establish temporality and directionality. Second, recruitment was limited to Wuhan, limiting generalizability to other regions or rural settings in China. Third, potential mediators—such as cognitive function and social support—were not assessed, leaving their role in the depression–PAU pathway unexamined. Fourth, perceived stress was measured globally; future investigations should incorporate chronic and domain-specific stressors to more precisely delineate stress–alcohol associations in older adults. Finally, all the data used in this study are self-reported, and related biases should be considered.

## 5. Conclusions

This study demonstrated a robust association between depressive symptoms and PAU among older Chinese adults. Multivariate logistic regression revealed that male sex, employment, and lower income were significant risk factors, reflecting sociocultural mechanisms such as gendered drinking norms, occupational drinking obligations, and socioeconomic stress. Network analysis revealed that anhedonia and sleep disturbance were the depressive symptoms most strongly and centrally connected to PAU. These findings underscore the need for targeted public health attention to socially vulnerable subgroups. Interventions enhancing reward processing (e.g., behavioral activation, reward-focused cognitive therapy) and circadian regulation (e.g., cognitive behavioral therapy for insomnia, CBT-I) hold promise for disrupting the bidirectional reinforcement between depression and PAU, offering clinically actionable pathways for prevention and treatment in this population.

## Availability of Data and Materials

The datasets used and analyzed during the current study are available from the corresponding author on reasonable request.
